# Development and Validation of an Endometriosis Diagnostic Method Based on Serum Biomarkers and Clinical Variables

**DOI:** 10.3390/biom13071052

**Published:** 2023-06-28

**Authors:** Bárbara Herranz-Blanco, Elza Daoud, Paola Viganò, Juan Antonio García-Velasco, Enrico Colli

**Affiliations:** 1 Chemo Research, 28050 Madrid, Spain; elza.daoud@exeltis.com (E.D.); enrico.colli@exeltis.com (E.C.); 2 Infertility Unit, Fondazione IRCCS Ca’ Granda Ospedale Maggiore Policlinico, 20122 Milano, Italy; paola.vigano@policlinico.mi.it; 3 Instituto Valenciano de Infertilidad (IVIRMA), 28013 Madrid, Spain; juan.garcia.velasco@ivirma.com

**Keywords:** endometriosis, biomarker, diagnosis

## Abstract

Endometriosis affects more than 10% of women of reproductive age, significantly impacting their quality of life. Diagnosis typically takes 4 to 11 years from symptom onset. The gold standard for diagnosing this disease, laparoscopy, is invasive, contributing to this delay in diagnosis. Two studies were conducted to develop a diagnostic test based on the combination of serum biomarkers and clinical variables. Study 1, the development study, aimed to: (i) confirm the ability of CA125, BDNF and clinical variables to differentiate between cases and controls, and (ii) develop a diagnostic algorithm based on these results. Study 2 validated the clinical performance of the developed in vitro diagnostic (IVD) test in diagnosing endometriosis. Serum samples and clinical variables extracted from psychometric questionnaires were obtained from the Oxford Endometriosis CaRe Centre biobank (UK). Case/control classification was performed based on laparoscopy and histological verification of the excised lesions. Studies 1 and 2 included *n* = 204 and *n* = 79 patients, respectively. Study 1 found a statistically significant difference between cases and controls for levels of both biomarkers. Of the assessed clinical variables from the patients’ medical histories, six were found to be significantly different between endometriosis cases and controls. CA125, BDNF and these six clinical variables were combined into a multivariable prediction model. In Study 2, the IVD test demonstrated sensitivity and specificity values of 46.2% (25.5–66.8%) and 100% (86.7–100%), respectively. Due to its high specificity, this IVD test is a simple and accurate rule-in test for early disease identification, even in the presence of non-specific symptoms.

## 1. Introduction

Endometriosis is an estrogen-dependent disease characterized by the growth of endometrial-like tissue outside the uterus [1,2]. These lesions cause a chronic inflammatory reaction, which can lead to the generation of scar tissue and adhesions [3]. Clinical symptoms include but are not limited to chronic pelvic pain, dysmenorrhea, dyschezia, dysuria, and infertility [4]. Endometriosis may increase a woman’s risk for chronic diseases such as cancer or autoimmune disorders, as well as overall morbidity [5,6,7]. Endometriotic lesions can occur in variable locations (both in and outside the pelvis), including the pelvic peritoneum and the ovary, and can infiltrate pelvic structures below the peritoneal surface (deep endometriosis). Three primary types of endometriosis have been defined based on their location: superficial peritoneal lesions (typically located on the pelvic organs or pelvic peritoneum), ovarian endometriomas, and deep infiltrating endometriosis (DIE) [8]. Endometriosis affects at least 10% of women of reproductive age. It is associated with a high societal and economic burden: the average annual cost of healthcare and loss of productivity due to pain from endometriosis was €9,579 for affected women from the United States and nine European countries [9].

Clinical examination does not reliably predict the presence of endometriosis in symptomatic women. Imaging techniques often fail to diagnose the disease, especially in early stages or when only superficial peritoneal lesions are present. However, imaging techniques can be useful in identifying endometriomas and, in some cases, deep endometriosis [1]. Regardless of the lesion type and location, the interpretation of imaging findings is highly dependent on a clinician’s experience and skill, hindering their utility [10]. Generally, when there is a high index of suspicion for endometriosis, women receive analgesics and hormonal medication without a definitive diagnosis [11]. Diagnosing the disease in these cases is only possible via an invasive laparoscopy with histologic confirmation of lesions [10]. This diagnostic hurdle contributes to the delay in definitive diagnosis for patients. On average, patients wait between 4 and 11 years between their first appearance of symptoms and their final definitive diagnosis [12]. Developing a non-invasive diagnostic tool is essential for faster diagnosis, selection of appropriate treatment, and triaging potential surgical patients [13,14].

Multiple biomarkers have been studied as screening and triage tests for endometriosis [15,16]. However, none have been implemented routinely in clinical practice [10]. In particular, Cancer Antigen 125 (CA125) has been extensively studied in endometriosis. CA125 is a high-molecular-weight glycoprotein expressed on the cell surface of some derivatives of embryonic coelomic epithelium, which are believed to be the precursors of endometriotic lesions [17]. Studies found CA125 levels to be higher in patients with endometriosis, indicating that CA125 can be a useful marker for diagnosing endometriosis, distinguishing the disease severity, monitoring the treatment effect, and identifying malignant transformation [18,19,20]. A meta-analysis on the diagnostic accuracy of CA125 for endometriosis pooling 22 studies, including 3626 participants, showed that CA125 performed well as a rule-in test. However, a negative test result is unable to rule out endometriosis. In addition, the study showed that CA125 was significantly more sensitive for diagnosing moderate or severe endometriosis (stages III and IV) compared with minimal disease [21,22,23]. Specifically, CA125 ≥ 30 U/mL was highly predictive of endometriosis in women with pain and/or subfertility symptoms, but CA125 < 30 U/mL could not rule out the disease [24].

Another biomarker of interest, brain-derived neurotrophic factor (BDNF), has been linked to several pathways disrupted in women with endometriosis. BDNF is a neurotrophin [25] with a high affinity to neurotrophic tyrosine receptor kinase 2 (NTRK2), also known as Tropomyosin receptor kinase B (TrKB). This ligand–receptor pair participates in some aspects of uterine physiology [26]. BDNF and NTRK2 expression was significantly increased in the uterus of women with endometriosis compared with disease-free controls [27]. Additionally, BDNF has been found to be overexpressed in ectopic but not in eutopic endometrial tissue. Interestingly, BDNF is a downstream effector of estrogens, mediating the pro-proliferative effects of estrogens promoting nociceptive pain [28,29]. Estrogens strongly induce BDNF production by macrophages, and BDNF promotes neurogenesis by binding to NTRK2 receptors on nerves. The release of pro-inflammatory mediators from mast cells, also triggered by estrogens, sensitizes peripheral nerve endings in endometriotic lesions, contributing to pain [30,31]. Several research groups have demonstrated that BDNF appears to be a reliable biomarker for early stages (I–II) of endometriosis [32,33,34].

Here, two studies aimed to develop a diagnostic tool that could identify all stages of endometriosis by combining CA125, BDNF and clinical variables. The development study (study 1) aimed to (i) confirm the ability of BDNF, CA125 and patients’ clinical information to differentiate between cases and controls and (ii) develop a diagnostic algorithm based on the results. The validation study (study 2) aimed to establish the clinical performance of the developed endometriosis IVD test, combining an ELISA kit to measure serum concentrations of BDNF and CA125 and a diagnostic software hosting the diagnostic algorithm.

## 2. Materials and Methods

### 2.1. Study Populations

Studies were conducted using serum samples and clinical data from the Oxford Endometriosis CaRe Centre biobank (UK). The CaRe Center biobank investigators selected the samples at random based on the inclusion and exclusion criteria established in the study protocols. This biobank emerged from the World Endometriosis Research Foundation (WERF) Endometriosis Phenome and Biobanking Harmonisation Project (EPHect) consensus on standardization and harmonization of phenotypic surgical/clinical data and biologic sample collection methods in endometriosis research. Patients included in this biobank were of reproductive age (18–50 years old) and were undergoing a laparoscopy due to suspicion of endometriosis. This biobank comprises serum samples, patients’ clinical information (from questionnaires) collected before surgery, and surgical information collected during the procedure. Patients were classified as controls and cases and anonymized in the biobank. Patients were classified as cases if endometriosis was confirmed by laparoscopy and histological evaluation of excised lesions and as controls if endometriosis lesions could not be visualized during the procedure or confirmed by histology. The patients with endometriosis were classified in stages after laparoscopy according to the revised American Society of Reproductive Medicine (rASRM) classification. Depending on imaging and surgical findings, endometriosis was further classified by lesion location: superficial, endometrioma, and/or DIE. Endometriosis was classified as “superficial” if superficial endometriosis lesions were only found on the ovaries or in the peritoneal cavity. Endometriosis was classified as “endometrioma” if endometriomas were found on the ovaries with or without superficial endometriosis. Endometriosis was classified as “DIE” if infiltrative lesions were reported in the peritoneal cavity with or without superficial endometriosis. Endometriosis was classified as “endometrioma + DIE” if DIE was found in the peritoneal cavity along with endometriomas (with or without superficial endometriosis). For 5 patients, this classification was not available. The Ethics Committee of CEIm HM Hospitals approved the experimental protocols. Two cohorts of patients were studied.

#### 2.1.1. Development Cohort

Serum samples from *n* = 204 patients were included in the development study: *n* = 136 patients with endometriosis and *n* = 68 controls. Table 1 shows the demographic characteristics of these patients. In this study, low- and high-stage endometriosis were equally represented in the cases group (stages I–II, 50%, and stages III–IV, 50%).

#### 2.1.2. External Validation Cohort

Serum samples from *n* = 79 patients were included in the validation study: *n* = 52 patients with endometriosis, and *n* = 25 controls. Table 2 shows the demographic characteristics of these patients. In this study, low-stage (I–II) endometriosis patients represented 81% of the cases.

### 2.2. Sample Collection

The specimens were collected and handled following the World Endometriosis Research Foundation standard operating procedures [35] after receiving the patients’ consent. Patients were asked to fast for at least 10 h before blood collection. Serum samples were stored in a biobank at −80 °C for up to 5 years and were transferred to the laboratory analysis site.

### 2.3. ELISA Method: CA125 and BDNF Concentrations

The IVD test ELISA (Enzyme-Linked Immunosorbent Assay) is a solid-phase sandwich enzyme-immunoassay for quantitatively determining BDNF and CA125 in human serum. Each biomarker was determined in a different set of wells. The ELISA plate was coated with an antibody directed against BDNF or CA125. BDNF or CA125 from samples and standards bound to the antibodies and were immobilized on the plate. Unbound biotin conjugate was washed off with washing solution. In a further step, streptavidin-HRP conjugate was added and bound to the biotin. Unbound streptavidin-HRP was washed off with washing solution. Finally, a substrate solution was added, and the existing complex catalyzed the chemical reaction of the substrate into a colored chemical entity. The enzymatic color reaction was stopped after a predefined time period. The concentration of the colored chemical correlating proportionally to the concentration of the antibody was measured photometrically.

This analytical method was calibrated against the WHO Reference Reagent brain-derived neurotrophic factor (BDNF) (NIBSC code: 96/534) for BDNF and Architect CA 125 II assay from Abbott for CA125. Trueness of the ELISA test for the BDNF assay was evaluated by diluting the WHO Reference Reagent for BDNF (NIBSC 96/534) in sample diluted to ten different levels ranging from 5 to 90 ng/mL. Calculating the individual percentage deviation at each level showed an average deviation across the measuring range of 7.48% (CI95%: −0.5–14.4%). Trueness of the ELISA test for the CA125 assay was investigated by comparing the assay to a CE-marked reference method (ARCHITECT CA125 II assay from Abbott) since no international reference reagent is available. A total of 60 samples were analyzed, and an average bias of −7.35% (CI95%: −11.90% to −7.52%) was observed compared with the reference assay.

### 2.4. Software Input and Score Calculation

In the validation study, upon collection of all the essential input parameters (serum CA125, serum BDNF and clinical variables), these data were introduced by the laboratory technicians into the IVD test diagnostic medical software hosting the data treatment algorithm. The algorithm outcomes were calculated and classified as positive or negative depending on whether the value was above or below the threshold value, respectively.

### 2.5. Statistical Analysis

Statistical analyses were performed using the software R, version 4.1.3 (R Foundation for Statistical Computing, Vienna, Austria), blinded to the surgical and imaging findings. Normal distribution was checked using the Shapiro–Wilk test. Because the BDNF and CA125 levels did not follow a normal distribution, the Mann–Whitney U analysis was used to compare BDNF and CA125 values between cases and controls. Sample sizes were chosen so that the 95% confidence interval did not exceed 0.3 for sensitivity and specificity outcomes around the expected value. To evaluate the importance of including both BDNF and CA125 in a diagnostic model, three logistic regression models with CA125 and BDNF as predictors were generated: one comparing the controls with all the cases, one comparing the controls with low-stage disease (S1–S2), and one comparing the controls with high-stage disease (S3–S4). Upon generation of these models, the Akaike information criterion (AIC) was applied during backward stepwise regression to identify if BDNF and CA125 could identify endometriosis cases in the model.

Based on the results, CA125, BDNF and selected clinical variables were combined into a multivariable logistic regression model. Missing data were estimated by imputation: a threshold of 10% for each predictor was used as the maximum proportion of missing data for imputation. At each cut-off, the sensitivity and specificity were computed together with the 95% confidence interval (CI). To compare the performance of the different regression models, we used ROC (Receiver Operating Characteristic) curves (Delacour et al., 2005). These allow comparison of specificity (proportion of negatives, i.e., controls, correctly identified as negatives) and sensitivity (proportion of positives, i.e., endometriosis cases, correctly identified as positives) of different models for different cut-off values. The higher these curves’ AUC (Area Under Curve), the better the method. The maximum possible AUC is 1, which would indicate a perfect classifier. The Wilson score with continuity correction [36] was used to estimate 95% confidence intervals for accuracy, specificity, and sensitivity results. After selecting the most accurate model, the score was derived based on the final predictors and the corresponding regression coefficients. Rule-in cut-off and associated sensitivity were derived in the development cohort based on a specificity of ≥90%.

In total, 122 clinical variables were considered for inclusion in the multivariable diagnostic algorithm. Many of these clinical variables have a time component, showing a correlation with age at the time of surgery, either based on Pearson’s correlation coefficient (for numerical predictors) or through Mann–Whitney U (for binary categorical predictors) or Kruskal–Wallis (for multiclass categorical predictors) analysis. In addition, a threshold of 10% for each clinical variable was used as the maximum proportion of missing data for imputation. As a result, the predictors with significant missing data points and/or a significant correlation with age at time of surgery or a significant association with another candidate clinical variable were excluded from the multivariate analysis. Chi-squared and Cochran–Armitage tests were used to determine which categorical variables were most strongly associated with endometriosis. Mann–Whitney U analysis was used for numerical variables.

The validation study involved computing algorithm scores and corresponding outcomes using the IVD test software. A positive diagnosis was assigned when the score exceeded the predetermined cut-off, while a negative diagnosis was given when the score fell below the cut-off. Utilizing these outcomes, the primary performance parameters (sensitivity and specificity) and secondary performance parameters (accuracy and AUC) were determined and reported, along with their corresponding 95% confidence intervals. The primary performance parameters results were compared with the values of the acceptance criteria established in the development study to determine whether the device’s clinical performance meets the criteria, i.e., whether the device can adequately classify the study subjects as positive or negative for endometriosis. The sensitivity and specificity in the validation study should be at least at the lower limits of the sensitivity and specificity 95% confidence intervals in the algorithm development study. Because the prevalence of stage I-II in the validation study was significantly higher than in the development study (Chi-square = 18.06, *p* < 0.001), the outcomes in the validation were weighted to give equal representation to the low-stage and high-stage groups.

## 3. Results

### 3.1. Diagnostic Performance of CA125 and BDNF in Endometriosis

**Figure 1:**
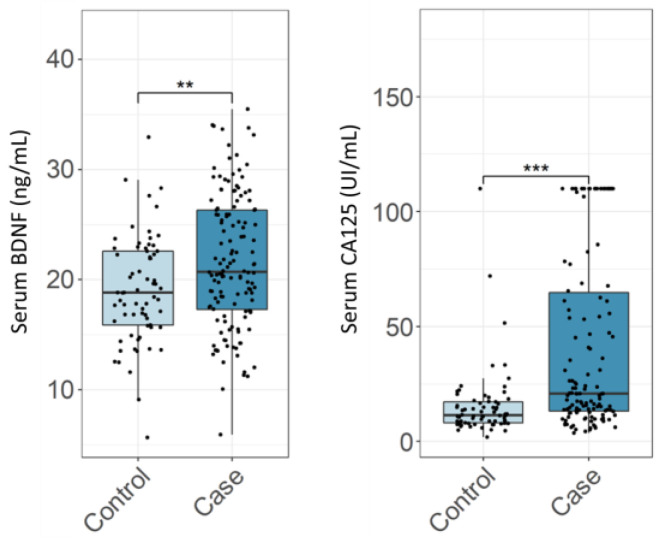
Serum concentration of CA125 and BDNF in endometriosis patients and controls. Asterisk signs above boxplots indicate a statistically significant difference in median value between the indicated population (**: *p* < 0.01; ***: *p* < 0.001) as established by a Mann–Whitney U test.

Both studied biomarkers, CA125 and BDNF, can distinguish endometriosis cases from controls with statistical significance (Figure 1). However, in backwards stepwise regression analysis based on AIC, for the comparisons of the control group with all cases and with the high-stage disease cases, both CA125 and BDNF were retained, meaning they were both independently informative as predictors of endometriosis. For the comparison of the control group with low-stage disease, only BDNF was retained, meaning that only BDNF was independently informative as a predictor of low-stage endometriosis.

Combined, CA125 and BDNF can distinguish controls from endometriosis cases with more accuracy than each biomarker independently, with the former performing very well in the high-stage group and the latter performing better in the low-stage group. Therefore, inclusion of both parameters in a multivariable model for endometriosis was justified. As the biomarkers performed well in distinct endometriosis stages, no individual cut-off values for each biomarker were determined.

### 3.2. Development of Prediction Model for Endometriosis

Among all the clinical variables related to a patient’s medical history that were considered, chi-square analysis showed that only three qualitative variables were significantly different between cases and controls. Most significantly, a history of previous exploratory surgery for endometriosis (even if the disease was not diagnosed) was more common among the patients who were diagnosed with endometriosis (54.6% in cases, 10.6% in controls, *p* < 0.001). Painful periods as a symptom leading to a referral for endometriosis was also associated with a positive diagnosis with strong statistical significance (76.4% in cases, 36.8% in controls, *p* < 0.001). Another significant variable was the severity of the last menstrual cycle pain, with moderate/severe pain being more frequent in cases than in controls (78.0% in cases, 47.1% in controls, *p* < 0.01). For numerical (quantitative) variables, the median of three of them were significantly different between cases and controls in the Mann–Whitney U test: age at first regular use of painkiller (U = 343.5, *p* = 0.038), age at first diagnosis of ovarian cyst (U = 334.5, *p* = 0.023), and age at first experience of intercourse pain (U = 1201, *p* = 0.009).

In the final revised model, the eight variables discussed above were considered: CA125, BDNF and the six clinical variables, i.e., record of previous surgery to examine for endometriosis, painful periods as a symptom leading to referral for endometriosis, the severity of menstrual pain during last cycle, age at first experience of intercourse pain, age at first regular use of painkillers and age at first diagnosis of ovarian cyst.

To estimate the test’s performance on independent data, a logistic regression model was repeatedly generated on 80% of the data and evaluated on the remaining 20%. The final model, generated from all algorithm development data, was optimized for high specificity to render a rule-in test with a low rate of false positives (Table 3) [37]. This model has an AUC of 0.867 with a sensitivity of 51.5% at a specificity of 95.6%.

### 3.3. Clinical Performance Evaluation (Validation of the IVD Test)

The diagnostic performance of the IVD test, comprising the ELISA kit method for determining BDNF and CA125, and the diagnostic algorithm established in the development study, was evaluated in a different sample cohort. The endometriosis IVD test had a sensitivity (after weighting for disease stages) of 46.2% (95% CI: 25.5–66.8%) and a specificity of 100% (95% CI: 86.7–100%). The accuracy was 64.1% (95% CI: 50.4–77.8%), and the AUC was 0.758 (95% CI: 0.650–0.867). With an observed diagnostic specificity in this clinical performance study of 100%, the target specificity of 86.8% (or higher) is met. A good specificity was the primary objective because this assay is primarily intended to aid in identifying individuals with endometriosis. For the sensitivity, a mid-range sensitivity rather than a low sensitivity was desired to ensure that a significant proportion of the test population will test positive.

### 3.4. Differential Diagnosis

#### 3.4.1. Confounding Conditions

Furthermore, we investigated whether other gynaecological conditions could interfere with the performance of the IVD test, rendering a positive test result when endometriosis is not present (false positive). The conditions considered as potentially confounding were non-endometriosis benign ovarian cysts, ovarian cancer, uterine fibroids and adenomyosis. In this respect, out of 93 controls included in the development (*n* = 68) and validation (*n* = 25) studies, 42% (*n* = 39) had ovarian cysts, 11% (*n* = 10) had uterine fibroids, 1% (*n* = 1) had adenomyosis and 1% (*n* = 1) had ovarian cancer. Only two patients (2%), one with ovarian cysts and one with uterine fibroids, had a positive test result (false positive); thus, the effect of these potentially confounding conditions on the test is minimal.

#### 3.4.2. Detection of Superficial Endometriosis

We analyzed the capacity of the endometriosis IVD test to identify the cases presenting just with superficial endometriosis. In the development cohort, endometriosis could be classified into different groups (superficial endometriosis, endometrioma, endometrioma + DIE and DIE) for 134 out of 136 cases. The classification could be performed for 50 out of 53 cases in the validation cohort. Out of the *n* = 184 patients in total (both cohorts), *n* = 79 patients had superficial endometriosis (43%). The endometriosis IVD Test detected *n* = 25 of the *n* = 79 (32%) cases with superficial endometriosis.

## 4. Discussion

We developed and validated an in vitro diagnostic (IVD) test for endometriosis. In the development study, the ability of BDNF and CA125 to differentiate between cases and controls was confirmed. Based on those results, the IVD test, consisting of an ELISA kit for determining serum concentrations of BDNF and CA125 and a data treatment algorithm hosted in diagnostic medical software was developed. The validation study established the clinical performance of the IVD test in diagnosing endometriosis. The main results are discussed below.

First, although no individual cut-off values were set, CA125 and BDNF levels were demonstrated to be elevated in patients with endometriosis, with CA125 mostly able to identify high-stage endometriosis and BDNF performing well for both low- and high-stage disease. This confirms what was previously found by other research groups: BDNF concentrations are higher in endometriosis patients than in controls in plasma [33,38,39] and serum [32,34,40]. We chose to measure BDNF concentration in serum because, as previously shown, all of the BDNF content is released from platelets during centrifugation, reducing the measurement errors related to blood handling, storage, and analysis encountered with plasma samples [41,42]. Although there is confounding evidence on the validity of CA125 as a biomarker for endometriosis, two meta-analyses previously showed that it could be used in conjunction with clinical information [20,23].

Several controls in both development and validation studies had other gynecological conditions that could elevate the CA125 concentration in serum (benign ovarian cysts, uterine fibroids and ovarian cancer) [22] and had a negative diagnosis (classified as true negative) using the IVD test. Such confounding factors did not lead to any false positive results in the validation study. This is likely because the IVD test relies not solely on CA125 but also BDNF and the patient’s clinical information.

In the validation study, as the algorithm was optimized for specificity during development, the novel endometriosis IVD test showed a limited sensitivity (46.2%) but a very high specificity of 100%, making it an excellent rule-in test able to minimize the risk of false positives. A rule-in test is considered the most appropriate approach given the chronic and non-life-threatening nature of the disease. A positive test result would aid the clinician in the diagnosis when considered together with other clinical information. The diagnosis of women presenting only with superficial lesions is of special interest due to the limited value of existing imaging techniques for their identification [1,10], possibly leading to underdiagnosis and numerous misdiagnoses. Considering that the endometriosis IVD test was able to detect 32% of cases presenting with superficial lesions recruited in the studies, this diagnostic tool can provide added value for the diagnosis of this type of disease. When the test is negative, the clinician may consider other causes for the symptoms or symptomatic treatment for pain, according to their usual practice. If the suspicion of endometriosis persists after a follow-up consultation, the woman can be re-tested at the clinician’s discretion.

Our diagnostic test compares well with other benchmark diagnostic tests such as prostate-specific antigen (PSA) to detect prostate cancer, which has a sensitivity of 93% (95% CI 88%, 96%) and a specificity of 20% (95% CI 12%, 33%) [43].

An essential strength of this study is that all the participants underwent laparoscopy (gold standard diagnosis) as a necessary component of the algorithm development is to provide the actual clinical state of each participant. The diagnostic algorithm was developed based on *n* = 204 patients in the development cohort. A total of eight predictors were included in the multivariate logistic regression model: CA125, BDNF, a record of previous surgery for endometriosis, painful periods leading to referral for endometriosis, age at first intercourse pain, age at first painkillers use, age at first ovarian cyst symptom and severity of menstrual pain during last cycle. After performing the IVD ELISA test, laboratory technicians can introduce CA125 and BDNF results into the diagnostic medical software along with patients’ medical information. The software hosting the algorithm calculates a score and provides a diagnosis based on predetermined cut-off values.

This novel endometriosis IVD test is a medical device that has been CE-marked under the IVD Directive 98/79/EC. The test could be included in the early workup to aid clinicians in diagnosing endometriosis when the disease is suspected, in conjunction with other clinical information, to facilitate timely access to correct disease management. Its simplicity makes it accessible to all healthcare providers, allowing general practitioners to detect the disease and refer patients to specialists early in the disease course.

## 5. Conclusions

We have developed and validated an in vitro diagnostic test for endometriosis. The excellent rule-in performance of this test could provide significant value in the clinical management of this disease. It is simple to use and can be performed by any health provider even in non-specialized settings.

## 6. Patents

There is a patent resulting from this work.

## Figures and Tables

**Table 1:** Demographic characteristics of the patients in the development cohort.

	Controls	Cases
	*n* = 68	*n* = 136
Age years (mean ± SD)	33.5 (5.96)	35.6 (6.42)
BMI (mean ± SD)	25.38 (4.63)	26.46 (5.32)
rASRM classification		
I–II	-	68 (50%)
III–IV	-	68 (50%)
Endometriosis classification		
Superficial	-	54 (39.7%)
Endometrioma	-	26 (19.1%)
DIE	-	29 (21.3%)
DIE + endometrioma	-	25 (18.4%)
Unclassified	-	2 (1.5%)
Other gynaecological conditions		
Ovarian cysts	28	66
Ovarian cancer	1	6
Uterine fibroids	7	25
Adenomyosis	0	7

Note: BMI = body mass index; rASRM = revised American Society for Reproductive Medicine; DIE = deep infiltrative endometriosis.

**Table 2:** Demographic characteristics of the patients in the external validation cohort.

	Controls	Cases
	*n* = 25	*n* = 52
Age years (mean ± SD)	35 (6.44)	35 (6.47)
BMI (mean ± SD)	26 (5.23)	26 (5.14)
rASRM classification		
I–II	-	42 (81%)
III–IV	-	7 (13%)
Missing information		3 (6%)
Endometriosis classification		
Superficial	-	25 (48.1%)
Endometrioma	-	3 (5.8%)
DIE	-	14 (26.9%)
DIE + endometrioma	-	8 (15.4%)
Unclassified	-	3 (5.8%)
Other conditions		
Ovarian cysts	11	16
Ovarian cancer	0	4
Uterine fibroids	3	4
Adenomyosis	1	1

Note: BMI = body mass index; rASRM = revised American Society for Reproductive Medicine, DIE = deep infiltrative endometriosis.

**Table 3:** Performance characteristics of the IVD test in the development study.

Model	Area under Curve	Youden’s Index	Accuracy	Sensitivity	Specificity
At 95% specificity	0.867	47.1%	66.2%	51.5%	95.6%
	(0.819–0.915)	(37.3–56.8%)	(59.2–72.5%)	(42.8–60.1%)	(86.8–98.9%)
At 95% sensitivity	0.867	44.1%	79.9%	95.6%	48.5%
	(0.819–0.915)	(31.7–56.5%)	(73.6–85%)	(90.2–98.2%)	(36.4–60.9%)
At maximum Youden’s index	0.867	58.8%	82.4%	88.2%	70.6%
	(0.819–0.915)	(46.7–70.9%)	(76.3–87.2%)	(81.3–92.9%)	(58.1–80.7%)
At maximum accuracy	0.867	58.1%	82.4%	89%	69.1%
	(0.819–0.915)	(45.9–70.3%)	(76.3–87.2%)	(82.2–93.5%)	(56.6–79.5%)

## Data Availability

The data presented in this study are available on request from the corresponding author. The data are not publicly available due to privacy.
